# Developmental asymmetry of the clavicles and third molars and its implications for forensic age estimation

**DOI:** 10.1038/s41598-025-28730-y

**Published:** 2025-11-21

**Authors:** Nina Heldring, Ali-Reza Rezaie, Ola Kvist

**Affiliations:** 1 Department of Forensic Medicine, Swedish National Board of Forensic Medicine, Blombäcks Väg 5, 171 65 Stockholm, Sweden; 2 Department of Laboratory Medicine, Centre for Biomolecular and Cellular Medicine, Karolinska Institutet, 141 86 Stockholm, Sweden; 3 Department of Women’s and Children’s Health, Karolinska Institute, Stockholm, Sweden; 4 Department of Radiology, Columbia University Irving Medical Center, New York, USA

**Keywords:** Age assessment, Asymmetry, Skeletal and dental, Chronological age, Forensic medicine, Developmental biology, Anatomy, Medical research

## Abstract

Age estimations are relevant for pre-trial detention and sentencing in criminal cases and as part of the evaluation in asylum processes to protect the rights and privileges of minors. No method can determine an exact chronological age due to individual variations in biological development. Current techniques assess skeletal or dental development and compare to reference populations. A key question is whether both sides of a body part need imaging, especially when asymmetric development occurs. This study evaluates whether bilateral imaging of the clavicles and third molars is necessary or if unilateral imaging suffices. We retrospectively analyzed clinical and radiological data from patients who underwent CT scans at Karolinska University Hospital, along with third molar data from studies using plain radiographs to assess development in relation to chronological age. The primary aim of this study is to examine the frequency of asymmetrical maturation in the medial clavicle and third molar in males and in females. The secondary aim was to examine how asymmetry influences age estimation in medico-legal contexts. To mitigate potential bias from relying on a single-reviewer assessment, we introduced a predefined level of misclassification into our model. Our findings show a strong correlation between right and left clavicle development (*ρ* = 0.871 (males) and *ρ* = 0.854 (females)) and near-perfect correlation (*ρ* = 0.980 (males) and *ρ* = 0.975 (females)) for third molars in both sexes. Asymmetrical development was found in approximately 23% (clavicle) and 13% (third molar) of males, and 20% and 17% of females, respectively. We recommend bilateral clavicle assessment to capture developmental variation and improve accuracy. For third molars, using the side with the most mature development in males and the least mature side in females enhances accuracy around the 18-year threshold.

## Introduction

Age estimations are relevant for pre-trial detention and sentencing in criminal cases as well as part of the evaluation in asylum processes to protect the rights and privileges of minors. No method currently used for medical age estimations can determine an exact chronological age due to the individual variations in biological development. The absence of accepted standardized methodologies has led to the adoption of varied approaches for age assessment across different countries^1,2^. Expert opinions for forensic age assessments in Germany have introduced a minimum-age concept which is designed to prevent the erroneous classification of minors as legal adults^3^, including the use of computer tomography (CT) examination of the clavicle^4^. However, one problematic consequence is that this approach is not efficient in distinguishing adults from minors and that large reference populations are missing.

The Swedish National Board of Forensic Medicine has developed a statistical model for estimating an age relative to key legal thresholds (15, 18, and 21-years) based on developmental stages of different parts of the skeleton, i.e. computer tomography (CT) of the clavicle, radiograph (RG) of the hand/wrist, magnetic resonance imaging (MRI) of the knee and teeth (RG of the third molar)^5^. Probability methods provide a most likely age distribution based on large reference populations together with error rates rather than an indeterminable chronological age (CA). The overall approach of the Swedish National Board of Forensic Medicine model for estimating the probability that an individual is below or above a certain age begins with assessing the developmental stages of a selected skeletal component together with the wisdom tooth. These observations are then compared to the age distribution in a large reference population of the same sex and corresponding developmental stages. The probabilities are supplemented with the margin of error for the final assessment, represented by the minor portion of the reference population distribution in relation to the chosen age threshold^5^. The ossification of the medial epiphysis of the clavicle occurs later than other skeletal elements and is therefore of particular interest when assessing the 21 year threshold^5–8^. The development of the wisdom tooth according to Demirjian stages spans from early childhood to the mid-twenties, making it applicable for assessing multiple age thresholds^9^. However, a critical consideration is determining the appropriate reference side for each body part and addressing potential developmental asymmetries between the right and left sides. Varying degrees of developmental asymmetry in the clavicles have been reported in previous studies^6,10–13^. Conversely, several studies have concluded that they find no significant difference between the development of left and right third molars^14–18^. This study investigates whether imaging both clavicles and third molars is necessary or if unilateral imaging is sufficient. Unilateral imaging would reduce radiation exposure, but may compromise diagnostic accuracy if asymmetry is present. The primary aim of this study is to examine the frequency of asymmetrical maturation in the medial clavicle and third molar in males as well as in females. A secondary aim is to evaluate the impact of this asymmetry on age estimation^6^in a medico-legal context.

## Methods

### Clavicle study cohort

We retrospectively evaluated the clinical and radiological data of patients who underwent CT examinations as part of routine clinical practice.at the Karolinska University Hospital. The population of males (*n* = 198) and females (*n* = 201) with a known sex and chronological age between 17.0 and 25.0 years of age were randomly selected from the database. Exclusion criteria included uncertain age, injury in the clavicle area or clavicle not visible, known disease and medications that may affect clavicle development, poor image quality or age other than 17.0–25.0 years at the time of examination. The CT scans were performed on either GE Revolution (GE Healthcare, Milwaukee, WI, USA) or Siemens Force (Siemens Healthineers, Erlangen, Germany) computed tomography scanners. The data was reformatted with bone kernel and voxel size of 0.6 × 0.6 × 0.6 mm. The medial clavicle was viewed in axial and coronal plane using a standard PACS workstation. Selected scans were subsequently assessed for developmental stage using the Schmeling staging system^6,19^ on the left and right side (5 stages) by one radiologist with 14 years of musculoskeletal (MSK) radiology experience and 8 years with focus on pediatric MSK radiology experience as well as age estimations from a medico-legal perspective. The final dataset included information on sex and chronological age together with development stage of both clavicles on an individual level. The protocol was approved by the Swedish Ethical Review Authority (Dnr 2024–00,531-01).

### Retrospective data-collection of third molar population

The sample consisted of 2567 individual assessments from orthopantomographs of females (*n* = 1347) and males (*n* = 1220) between 17 and 25 years and of American (Texas) or European origin. The third molar dataset was retrospectively collected and originated from studies relating the development of the third molar in the lower jaw, imaged with plain radiographs and classified by Demirjian (8 stages) to chronological age. Three datasets, two of which have been published, qualified after the selection process requiring classification of both right and left third molar in the same individuals with a known exact chronological age. The three datasets have been provided by authors and researchers upon contact^20,21^.

### Statistical analysis

To calculate the minimal sample size needed for the clavicle cohort, the same approach was used as described previously^5^. Briefly, a prediction model with a binary outcome (correct or incorrect classification)^22^including a conservative outlook with a c-statistics of 0.85 and a prevalence of 0.15, meaning 15% misclassification of events are expected. This resulted in 195 individuals for a sample size for males and females, respectively.

The Spearman correlation coefficient was used to assess the relationship between the left and right sides of the clavicle^23^, as well as between the left and right third molars (tooth 38 and tooth 48, respectively). Individuals with missing values for either side of the clavicle or third molar were excluded from the correlation analysis, ensuring that only complete observations were used. A two-proportion Z-test was conducted to assess whether the proportions differed between the left and right sides of the clavicle and third molar in subjects with different developmental stages on either side. The Receiver operating characteristic (ROC)-curves were generated in R with the *pROC* package^24^. All other analyses, data management and figure generation were conducted in R (version 4.3.1)^25^using the packages *caret*, *lattice, ggplot2* and *ggpubr*^26–29^.

Binary classification of being over and under a certain age threshold and consequently correctly or incorrectly classified was assessed by using the probability model presented in Heldring et al. (2024)^5^. The obtained probabilities for the clavicle or third molar was used for a ROC-curve analysis^30^ to maximize sensitivity (correctly identifying individuals above the age threshold) and specificity (correctly identifying individuals below the age threshold). Furthermore, this analysis was done on the estimated maturation stage of either left, right, most or least mature side. The cut-off point of the ROC-curve analysis was set to the probability of 0.35 for both the clavicle and third molar for both the 18 and 21 year-old threshold.

## Results

Clavicle development was evaluated bilaterally using CT images from 399 individuals (198 males and 201 females) with an age range of 17.0–25.0 years (Table 1 a-b). The populations are evenly distributed over the age cohorts in both sexes and included individuals in all 5 stages according to the Schmeling scale^6,19^. The least mature stage (stage 1) is observed up to the age of 17 in females and 21 in males while few individuals below 25 reached the most mature stage (stage 5) (Table 1 a-b). The third molar populations are compiled from previous studies and included 2567 individuals (1220 males and 1347 females) (Table 2 a-b). This population has an approximately normal distribution over the age cohorts 8–26 year (males) and 7–25 years (females) and with a mean age of 16.94 for females and 16.71 for males, covering all eight Demirjian stages^9^. The least mature stage (A) is observed up to 13–14 years of age and the minimal age for most mature stage (H) is observed in 17-year-old males and females although more common in individuals older than 20 years (Table 2 a-b).

**Table 1:** Age distribution for clavicle in (a) males and (b) females.

Clavicle											
Stage		Stage 1		Stage 2		Stage 3		Stage 4		Stage 5	
Age	No of males	Left	Right	Left	Right	Left	Right	Left	Right	Left	Right
17	22	5	3	15	18	2	1	0	0	0	0
18	22	3	1	14	18	5	3	0	0	0	0
19	22	0	1	16	15	6	6	0	0	0	0
20	22	0	0	10	11	10	9	2	2	0	0
21	21	0	1	4	3	14	15	3	2	0	0
22	23	0	0	2	6	12	19	9	7	0	0
23	22	0	0	0	0	10	10	12	12	0	0
24	22	0	0	0	0	9	8	13	13	0	1
25	22	0	0	0	0	3	3	17	17	2	2
Sum	198	8	6	61	71	71	65	56	53	2	3

Clavicle											
Stage		Stage 1		Stage 2		Stage 3		Stage 4		Stage 5	
Age	No of females	Left	Right	Left	Right	Left	Right	Left	Right	Left	Right
17	22	1	2	16	17	5	3	0	0	0	0
18	22	0	0	12	15	10	6	0	1	0	0
19	24	0	0	9	9	14	15	1	0	0	0
20	23	0	0	8	9	13	13	2	1	0	0
21	22	0	0	4	4	13	12	5	6	0	0
22	22	0	0	0	1	16	15	6	6	0	0
23	22	0	0	0	0	8	8	14	13	0	1
24	22	0	0	0	0	6	7	16	14	0	1
25	22	0	0	0	0	2	1	20	18	0	3
Sum	201	1	2	49	55	87	80	64	59	0	5

**Table 2:** Frequency table for third molar in (a) males and (b) females.

Third molar																	
Stage		A		B		C		D		E		F		G		H	
Age	No of males	Left	Right	Left	Right	Left	Right	Left	Right	Left	Right	Left	Right	Left	Right	Left	Right
8	14	11	11	3	3	0	0	0	0	0	0	0	0	0	0	0	0
9	30	7	11	18	14	5	5	0	0	0	0	0	0	0	0	0	0
10	52	4	7	25	24	18	17	4	4	1	0	0	0	0	0	0	0
11	75	2	4	20	19	31	31	21	20	1	1	0	0	0	0	0	0
12	94	1	2	15	14	37	35	35	35	6	8	0	0	0	0	0	0
13	77	2	3	3	3	24	24	38	37	10	10	0	0	0	0	0	0
14	73	0	0	0	0	11	12	33	34	24	22	5	5	0	0	0	0
15	121	0	0	0	0	5	7	48	44	48	49	20	21	0	0	0	0
16	95	0	0	0	0	2	3	13	11	34	35	39	36	7	10	0	0
17	112	0	0	0	0	0	0	8	6	20	24	46	45	31	31	7	6
18	99	0	0	0	0	0	0	2	2	6	7	22	22	44	39	25	29
19	83	0	0	0	0	0	0	0	0	4	4	6	3	26	30	47	46
20	85	0	0	0	0	0	0	0	0	0	0	2	2	20	24	63	59
21	73	0	0	0	0	0	0	0	0	2	2	1	1	12	15	58	55
22	79	0	0	0	0	0	0	0	0	1	1	0	1	10	10	68	67
23	22	0	0	0	0	0	0	0	0	0	0	0	0	4	2	18	20
24	18	0	0	0	0	0	0	0	0	0	0	0	0	3	2	15	16
25	17	0	0	0	0	0	0	0	0	0	0	0	0	0	0	17	17
26	1	0	0	0	0	0	0	0	0	0	0	0	0	0	0	1	1
Tot	1220	27	38	84	77	133	134	202	193	157	163	141	136	157	163	319	316

Third molar																	
Stage		A		B		C		D		E		F		G		H	
Age	No of females	Left	Right	Left	Right	Left	Right	Left	Right	Left	Right	Left	Right	Left	Right	Left	Right
7	3	1	1	2	2	0	0	0	0	0	0	0	0	0	0	0	0
8	10	2	1	7	8	1	1	0	0	0	0	0	0	0	0	0	0
9	22	6	6	13	13	3	3	0	0	0	0	0	0	0	0	0	0
10	46	9	10	25	24	6	6	6	6	0	0	0	0	0	0	0	0
11	66	3	3	17	16	21	23	24	24	1	0	0	0	0	0	0	0
12	103	5	3	14	16	33	34	45	43	6	7	0	0	0	0	0	0
13	89	1	2	7	7	19	16	48	46	11	15	3	3	0	0	0	0
14	84	1	0	1	2	20	18	33	39	28	21	1	4	0	0	0	0
15	126	0	0	0	0	11	10	53	51	45	47	13	16	4	2	0	0
16	122	0	0	0	0	2	3	28	23	45	49	37	34	10	13	0	0
17	122	0	0	0	0	1	0	7	8	28	29	49	49	29	30	8	6
18	122	0	0	0	0	0	1	11	9	17	15	36	44	40	32	18	21
19	95	0	0	0	0	0	0	2	2	9	7	19	18	31	30	34	38
20	94	0	0	0	0	0	0	3	2	7	4	17	16	32	40	35	32
21	93	0	0	0	0	0	0	0	0	5	3	7	5	21	26	60	59
22	93	0	0	0	0	0	0	0	0	0	1	3	3	15	16	75	73
23	22	0	0	0	0	0	0	0	0	0	0	2	1	7	10	13	11
24	16	0	0	0	0	0	0	0	0	0	0	0	0	3	3	13	13
25	19	0	0	0	0	0	0	0	0	0	0	0	0	0	4	19	15
Tot	1347	28	26	86	88	117	115	260	253	202	198	187	193	192	206	275	268

**Fig. 1:**
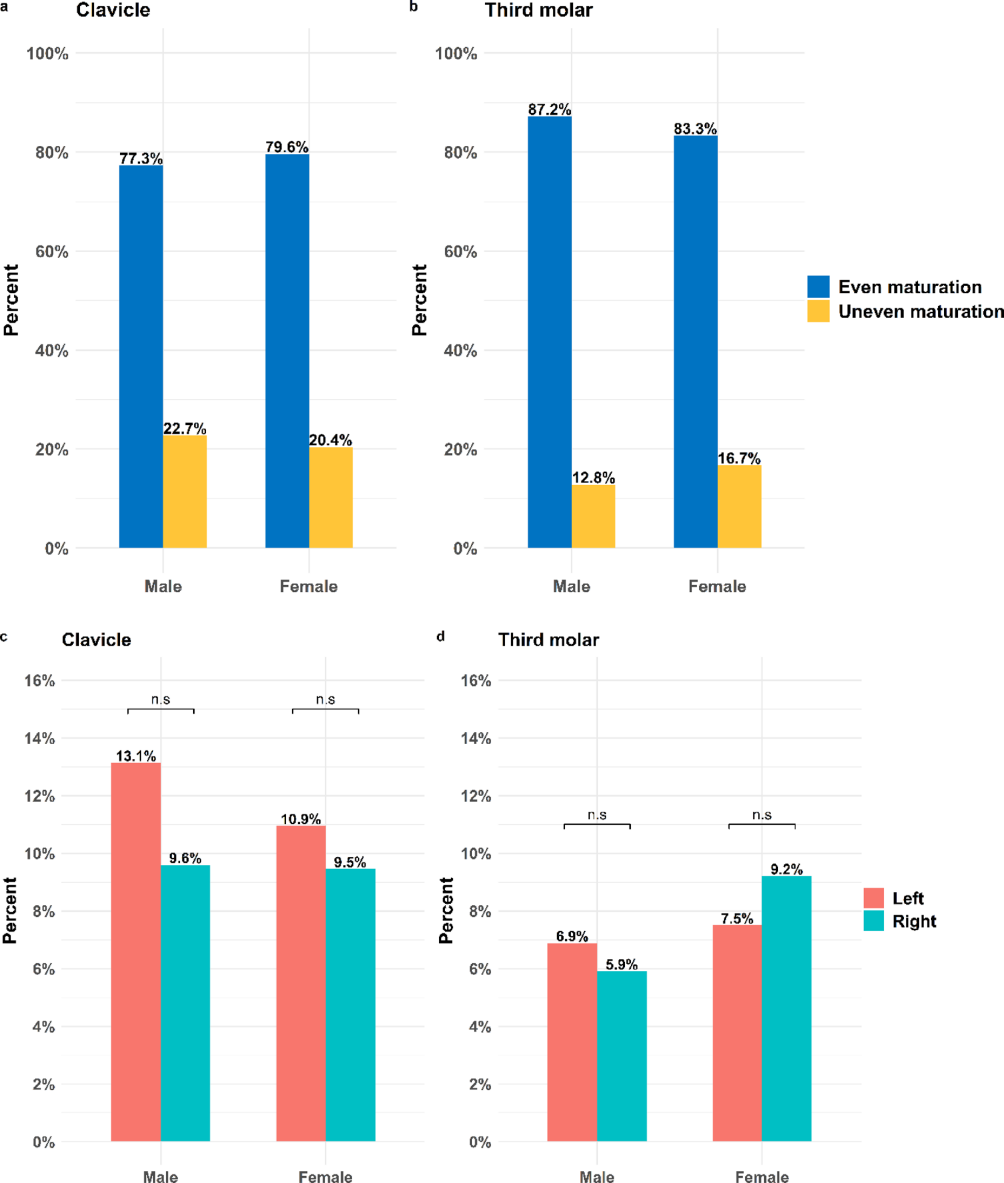
Maturation of right and left side in clavicle and third molar. Percent of even or asymmetrical maturation pattern in clavicle (**a**) or third molar (**b**). Percent of most mature side when asymmetrical maturation pattern is observed for clavicle (**c**) and third molar (**d**) in males and females.

Symmetric ossification of the clavicles was observed in 77% of males and 80% of females (Fig. 1a). Consequently, an asymmetrical (left and right side) clavicle-maturation is observed in approximately 23% of the males and 20% of females (Fig. 1a). Compared to clavicle, third molar development is more often observed to mature in an even fashion regarding left and right side, approximately 87% of the males and 83% of the women (Fig. 1b). The asymmetrical maturation (left and right side) is observed in 13% of the males and close to 17% of the women (Fig. 1b). Spearman’s rank correlation analysis showed a strong positive correlation between right and left side development of the clavicle and close to a perfect correlation in third molar for both males and females (Table 3).

**Table 3:** Spearman’s rank correlation coefficient between right and left side development in clavicle, or third molar.

Correlation		
Gender	Clavicle (*ρ*)	Third molar (*ρ*)
Male	0.871	0.980
Female	0.854	0.975
Overall	0.864	0.978

Among the male individuals, the left clavicle develops before the right side in 13% of the population and the corresponding percentage for the right clavicle is 10% (Fig. 1c). In females the left side was observed to develop before the right side in 11%, and the right side before the left side in approximately 9% (Fig. 1c). This means that the left side is predominantly the more mature side in 58% of the males and in 54% of the females, with an asymmetrical clavicle development (left and right). A two-proportion Z-test revealed no significant difference in the order of development between sides, neither in males nor females (Fig. 1 c). For third molar, the proportions of individuals with an asymmetrical maturation (left and right) are lower compared to clavicle (Fig. 1d). In addition, the asymmetrical maturation of left (7%) or right (6%) third molar being most mature is observed in a nearly equal proportion in males (Fig. 1d). In females, approximately 9% of the population demonstrate a more mature third molar on the right side while approximately 7.5% on the left side (Fig. 1d). The two-proportion Z-test revealed no significant difference (Fig. 1 d).

**Fig. 2:**
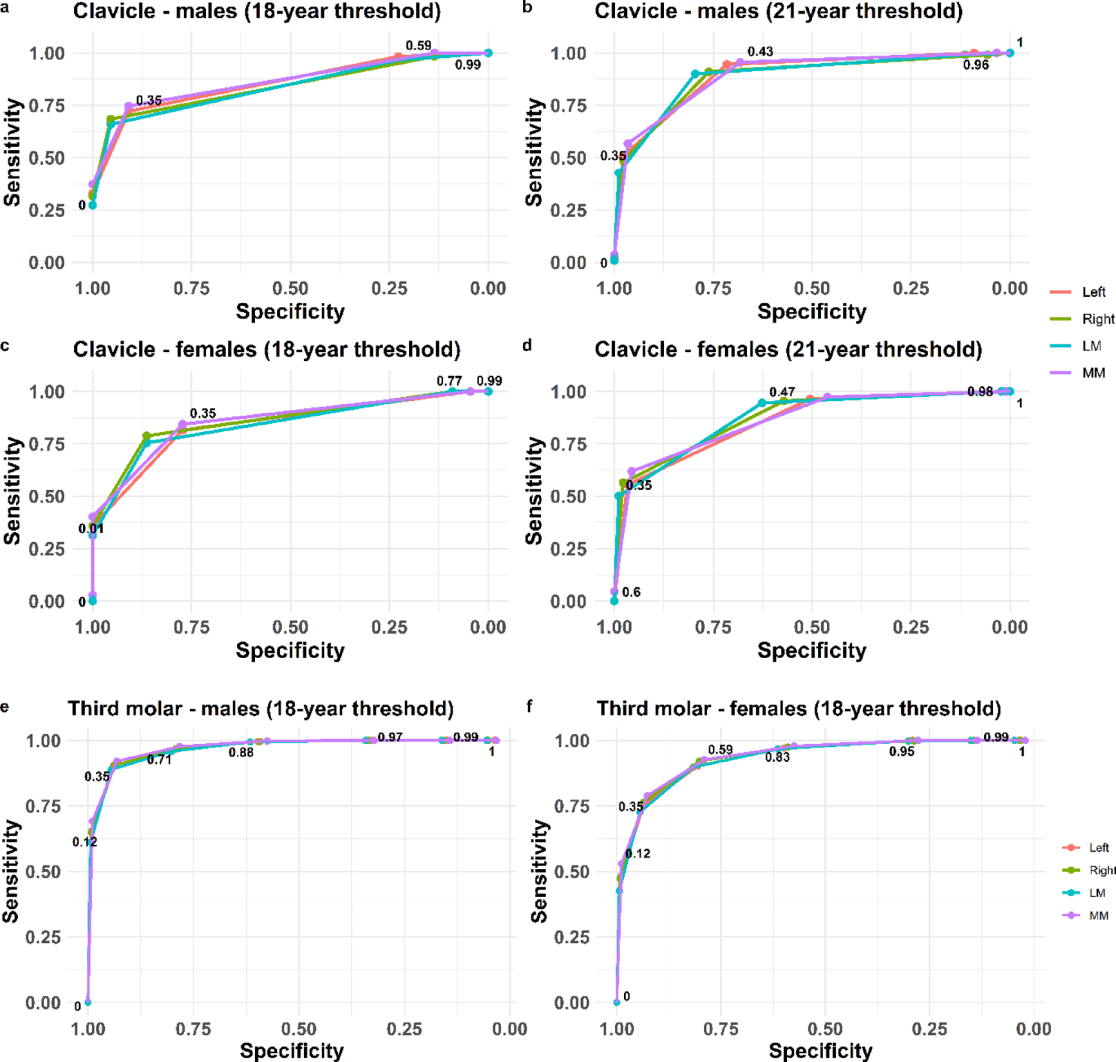
Calculated ROC-curves of left, right or most mature or least mature side for clavicle for the 18-year and the 21-year threshold in males (**a** and **b**) and females (**c** and **d**). The corresponding calculated ROC-curves for the 18-year threshold for third molar is shown for males (**e**) and females (**f**).

**Fig. 3:**
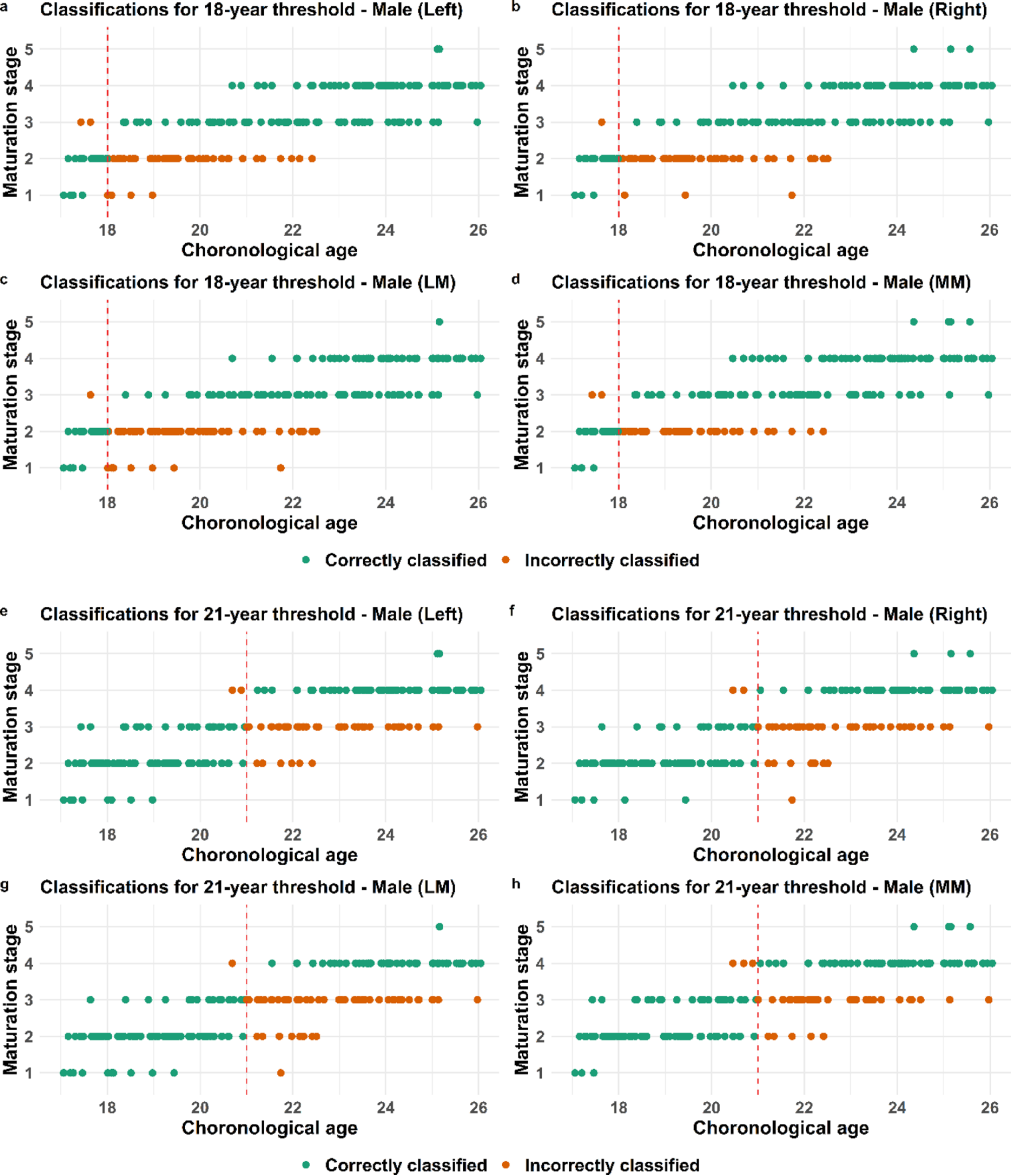
Classification of males based on clavicle development. Point plots displaying the chronological age and corresponding clavicle development stage for each individual together with classification, where green dots represent correctly classified and orange dots incorrectly classified males. Classification is done with regard to the 18 year threshold (**a**–**d**) or the 21 year threshold (**e**–**h**).

**Fig. 4:**
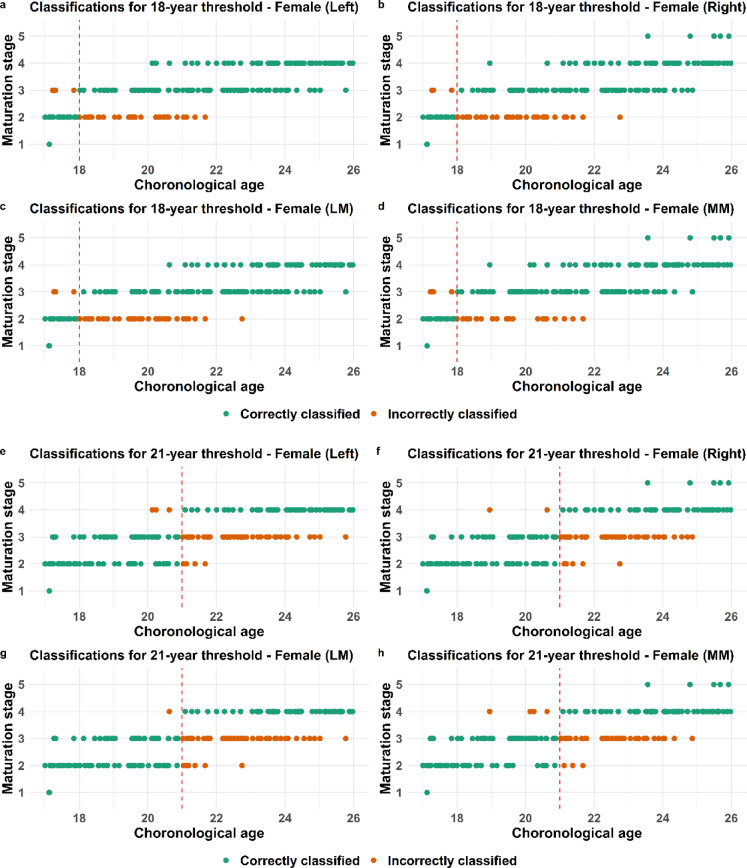
Classification of females based on clavicle development. Point plots displaying the chronological age and corresponding clavicle development stage for each individual together with classification, where green dots represent correctly classified and orange dots incorrectly classified females. Classification is done with regard to the 18 year threshold (**a**–**d**) or the 21 year threshold (**e**–**h**).

**Fig. 5:**
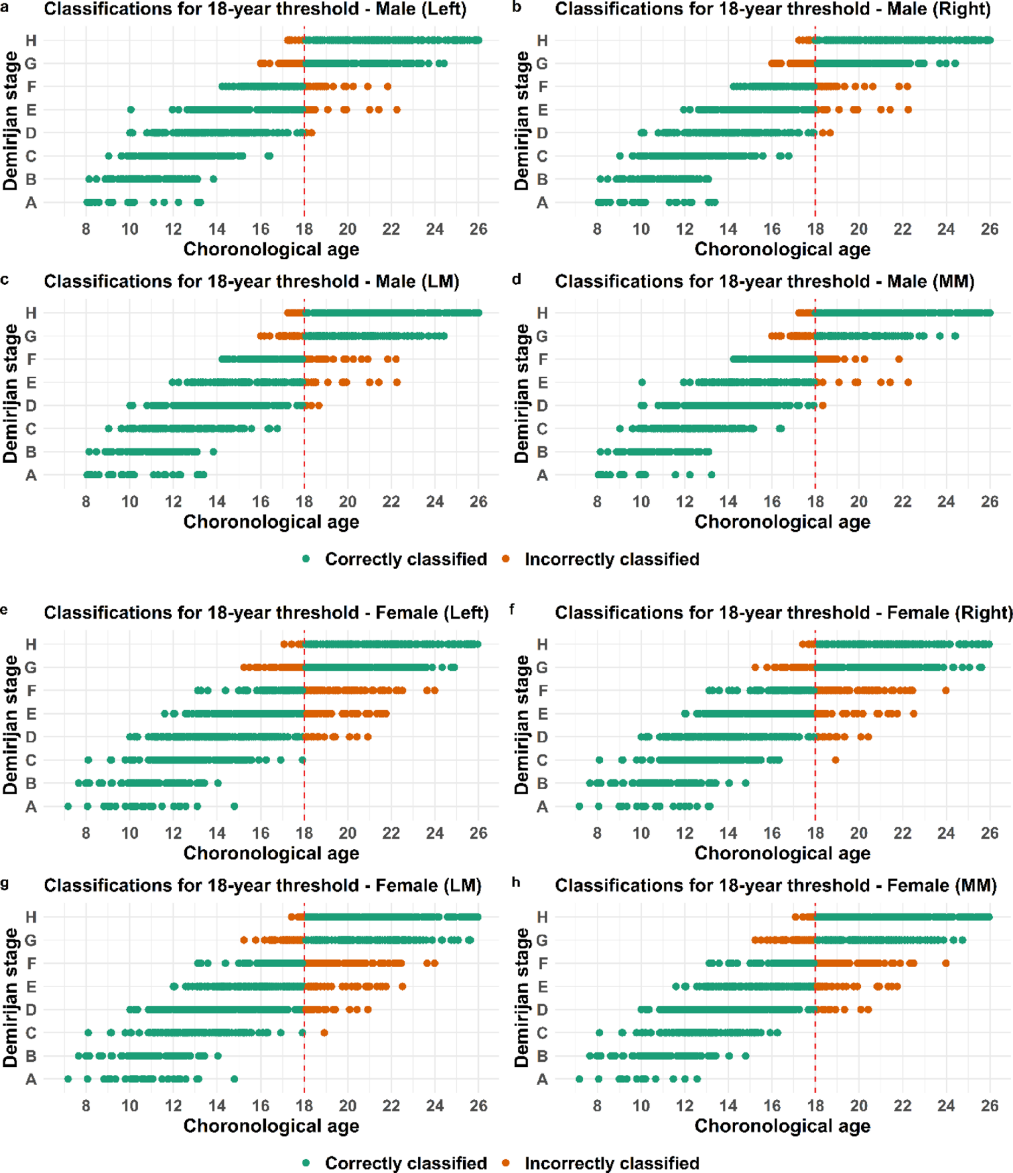
Classification based on third molar development. Point plots displaying the chronological age and corresponding third molar development stage for each individual together with classification, where green dots represent correctly classified and orange dots incorrectly classified individuals with regard to the 18 year threshold for males (**a**–**d**) or females (**e**–**h**).

A process to evaluate whether an individual is correctly classified as being below or above a certain age threshold involves selecting a cutoff point. Using a ROC curve analysis revealed that 35% is an optimal cutoff point to maximize sensitivity and specificity in both clavicle and third molar in males and females (Fig. 2 a-f). The corresponding total accuracy together with positive predictive value (PPV) and negative predictive value (NPV) as well as positive and negative likelihood ratios (LR + and LR-) were also calculated and these measures were indirectly maximized at the determined cut-off point. The validated statistical model for clavicular development is based on five primary stages, with substages excluded due to insufficient sample sizes^5^. Accordingly, the model utilizes the five main stages as defined by Schmeling^6^.The male and female individuals being correctly or incorrectly classified in the validated statistical model^5^based on the estimated maturation stage of either left, right, most (MM) or least mature (LM) side are visualized and presented in point-plots (Figs. 3, 4 and 5). The majority of individuals classified based on developmental stage are correctly classified (green dots) for both the clavicle and the third molar and this applies to both males and females (Figs. 3, 4 and 5).

The incorrectly classified male individuals (orange dots) in regard to the 18-year threshold are mainly in clavicle development stage 2 (Fig. 3 a-c). The least number of incorrectly classified individuals is observed when classified based on the most mature side (MM) which also applies to the 21 year threshold (Fig. 3 d-f). However, the incorrectly classified males are mainly in development stage 3 (Fig. 3 d-f), in regard to the 21 year threshold.

The incorrectly classified females (orange dots) are similar to males at development stage 2 concerning the 18-year threshold and stage 3 in relation to the 21 year threshold (Fig. 4 a–f). Visually, the lowest occurrence of orange dots, indicating the fewest misclassified females, is observed when classification is based on the most mature side (MM) for both age thresholds (Fig. 4 d–f). The incorrectly classified males (orange dots) based on third molar development are in stage E, F and G regarding the 18-year threshold (Fig. 5 a-d). For females, misclassification (orange dots) occurs across a broader developmental range, primarily from D to G (Fig. 5 e–h).

The quantitative measure analysis revealed that the most mature side provided the highest accuracy of age threshold assessment for both the 18 and 21 year threshold in clavicle (Table 4 a-b) using a recently published statistical model^5^. This applies to both males and females. For the 18 year threshold in third molar the most mature side provides the highest accuracy for males while the least mature side is favorable in females (Table 4 c).

**Table 4:** Quantitative reliability for clavicle for 18 and 21 year threshold in (a) males and (b) females and for third molar in males and females in (c) 18 year threshold.

Males, clavicle								
Measure	18 year threshold				21 year threshold			
	Left	Right	Least mature side	Most mature side	Left	Right	Least mature side	Most mature side
Sensitivity	0.722	0.684	0.659	0.746	0.509	0.486	0.427	0.568
Specificity	0.909	0.955	0.955	0.909	0.977	0.977	0.989	0.966
PPV	0.984	0.992	0.991	0.985	0.966	0.964	0.979	0.955
NPV	0.290	0.273	0.259	0.308	0.614	0.601	0.580	0.639
LR +	7.934	15.200	14.644	8.198	22.130	21.130	38.818	16.706
LR-	0.306	0.331	0.357	0.279	0.503	0.526	0.579	0.447
Accuracy	0.742	0.714	0.692	0.764	0.717	0.704	0.677	0.744

Females, clavicle								
Measure	18 year threshold				21 year threshold			
	Left	Right	Least mature side	Most mature side	Left	Right	Least mature side	Most mature side
Sensitivity	0.816	0.788	0.754	0.844	0.555	0.564	0.500	0.618
Specificity	0.773	0.864	0.864	0.773	0.967	0.978	0.989	0.956
PPV	0.967	0.979	0.978	0.968	0.953	0.969	0.982	0.944
NPV	0.340	0.333	0.302	0.378	0.642	0.650	0.621	0.674
LR +	3.595	5.794	5.544	3.718	16.818	25.636	45.455	14.045
LR-	0.238	0.245	0.285	0.202	0.460	0.446	0.506	0.400
Accuracy	0.811	0.796	0.766	0.836	0.741	0.751	0.721	0.771

18 year threshold. third molar								
Measure	Males				Females			
	Left	Right	Least mature side	Most mature side	Left	Right	Least mature side	Most mature side
Sensitivity	0.904	0.906	0.889	0.920	0.751	0.764	0.789	0.726
Specificity	0.939	0.937	0.945	0.931	0.936	0.936	0.927	0.945
PPV	0.905	0.902	0.912	0.896	0.891	0.892	0.883	0.901
NPV	0.938	0.939	0.930	0.948	0.843	0.850	0.863	0.831
LR +	14.820	14.381	16.164	13.333	11.734	11.938	10.808	13.200
LR-	0.102	0.100	0.117	0.086	0.266	0.252	0.228	0.290
Accuracy	0.925	0.925	0.923	0.927	0.860	0.865	0.870	0.854

Quantitative measures of the age assessment model’s reliability for the clavicle model (a) males and (b) females and (c) the third molar model. Quantifications based on independent dataset validations. Sensitivity (CA above age limit and identified as above the age limit), specificity (CA under age limit identified as under the age limit), positive predictive value ((PPV) individuals identified as above the age limit with CA above the age limit), negative predictive value ((NPV) individuals identified as below the age limit with CA below the age limit), positive likelihood ratio ((LR +) ratio that quantifies how much more likely a positive test result is to occur in individuals truly over 18 years of age than in those under 18), ((LR- ratio that quantifies how much less likely a negative test result is to occur in individuals truly over 18 years of age than in those under 18) and total accuracy presented for the 18 year and 21 year thresholds.

## Discussion

Similar to several other studies^6,8,10,11,31,32^, our findings demonstrate a strong correlation between the epiphyseal ossification of the clavicles and age. When determining whether an individual has reached the age threshold for criminal liability of 21 years, the clavicle is of particular interest, as other skeletal indicators have typically completed their maturation by this age. Similar to previous studies^10,11^, the least mature stage (stage 1) is in our study observed up to the age of 17 in females and 21 in males while few individuals below 25 has reached the most mature stage (stage 5). The probability of having mature clavicles at a certain age has been concluded to be affected both by the sex and socioeconomic status of the individual^33^. Our study shows a difference between left and right clavicular epiphyseal stages in approximately 23% of the males and 20% of females. Compared to clavicle, asymmetrical maturation of third molar (left and right side) is less frequent and is observed in 13% of the males and close to 17% of the women, which is in close agreement with a previous study^31^. Spearman’s rank correlation analysis revealed a strong positive correlation between the development of the right and left clavicle, as well as a near-perfect correlation for the third molar, in both males and females (Table 3). However, this test imposes a greater penalty on the correlation when the developmental stage difference between the right and left sides exceeds one stage. In our male and female populations, a difference of only one developmental stage between the right and left sides was observed, which did not lead to a significant reduction in the correlation coefficient. In line with our high Spearman correlation indicating symmetry, Angelopoulos et al. found no significant left and right difference in the lower third molar development, using a Wilcoxon signed-rank test^34^. An asymmetric clavicle development has been reported in the range of 0.4%–20.1% in previous studies^6,8,10–12^. The proportion of individuals with an asymmetric development in our study agrees with the rate of 21.0% reported in Bassed et al. from 2011 and 2012^11,31^. Similarly, Dreizen et al. found that only 26% of the children had identical skeletal ages of left and right hand/wrist and 22% had more advanced bone age of the left hand compared to the right^35^. In our study, the majority of the left/right differences were seen in stages III and IV which is in complete agreement with Bassed et al.^11^. It is unlikely that the asymmetric development is connected to the dominant side since the numbers in our study or any of the other studies are not in agreement with the proportion of approximately 10% of the population being left-handed. Historically, the non-dominant side was chosen to reduce the risk of older Salter-Harris fractures that might have affected the growth plate closure^36^. For the individuals with an observed asymmetric development, we do not detect any significant difference in the order of development between sides, neither in males nor females, indicating that it is driven by other developmental factors than dominancy.

Developmental asymmetry presents challenges for accurate age estimation, particularly in legal contexts. In attempting to provide an age estimate for a living individual who is the subject of a criminal investigation, and for whom the consequences will alter depending on whether they are over or under the age of 18 or 21 years, it is of great interest to reach as high accuracy as possible. We used a previously published statistical model^5^ to evaluate whether sensitivity, specificity, NPV, PPV, LR + , LR- as well as total accuracy is affected depending on an asymmetric development of either clavicle or third molar. The quantitative measure analysis demonstrated that the side exhibiting the most advanced developmental stage, whether left or right, yielded the highest total accuracy in determining age thresholds for both the 18 and 21 year thresholds with clavicular development. This observation was consistent across both male and female populations. These findings suggest that unilateral clavicle imaging may compromise age assessment accuracy. Therefore, it is recommended that both clavicles be imaged and assessed in order to achieve a more precise assessment of age in relation to age thresholds, particularly for legal purposes where age assessment is critical. By considering both clavicles, any potential asymmetry or variation in developmental progression between the sides can be accounted for, leading to a more robust and reliable age estimation. For third molar, the most mature side provides the highest accuracy for males while the least mature side is favorable in females for the 18 year threshold. A consensus in the field is that multifactorial age estimation performs better than that based on a single anatomical site^37–39^ and the combined dental and skeletal assessment allow increasing the accuracy even further.

### Limitations

The primary limitation is the single-reviewer assessment of clavicular ossification, which may introduce bias. The assessment of clavicular ossification is influenced by the individual assessor’s experience, training, and subjective interpretation. To account for the potential bias introduced by a single-reviewer assessment, we incorporated a predefined degree of misclassification into our model.

## Conclusion

Our study shows a strong positive correlation between right and left side development of the clavicle and close to a perfect correlation in third molar for both males and females. An asymmetrical clavicle or third molar-maturation is observed in approximately 23% or 13% respectively of the males and 20% or 17% respectively of females. We recommend bilateral clavicle assessment to capture developmental variation and when using a probability model, use the most mature side to improve accuracy. For third molars, using the side with the most mature development in males and the least mature side in females enhances accuracy around the 18-year threshold.

## Data Availability

Data may be obtained by contacting the first author. Email: nina.heldring@rmv.se.

## References

[1] EASO. EASO practical guide on age assessment. second edition ed: EU (2018).

[2] Committee SA. Biological evaluation methods to assist in assessing the age of unaccompanied asylum‑seeking children. In: office UH, ed. https://www.gov.uk/government/publications/2023.

[3] Schmeling, A., Dettmeyer, R., Rudolf, E., Vieth, V. & Geserick, G. Forensic age estimation. *Dtsch. Arztebl. Int.* **113**(4), 44–50 (2016). 26883413 10.3238/arztebl.2016.0044 PMC4760148

[4] Ruder, T. D. et al. Standards of practice in forensic age estimation with CT of the medial clavicular epiphysis-a systematic review. *Int. J. Legal. Med.* **137**(6), 1757–1766 (2023). 37691040 10.1007/s00414-023-03061-7 PMC10567934

[5] Heldring, N., Rezaie. A. R., Larsson, A., Gahn, R., Zilg, B. Camilleri S, et al. A probability model for estimating age in young individuals relative to key legal thresholds: 15, 18 or 21-year. Int J Legal Med. (2024). 10.1007/s00414-024-03324-x PMC11732925 39292274

[6] Schmeling, A. et al. Studies on the time frame for ossification of the medial clavicular epiphyseal cartilage in conventional radiography. *Int J Legal Med.* **118**(1), 5–8 (2004). 14534796 10.1007/s00414-003-0404-5

[7] Vieth V, Schulz R, Brinkmeier P, Dvorak J, Schmeling A. Age estimation in U-20 football players using 3.0 tesla MRI of the clavicle. Forensic Sci Int. 241:118–122 (2014). 10.1016/j.forsciint.2014.05.008 24908196

[8] Schulz, R. et al. Studies on the time frame for ossification of the medial epiphysis of the clavicle as revealed by CT scans. *Int. J. Legal Med.* **119**(3), 142–145 (2005). 15711799 10.1007/s00414-005-0529-9

[9] Demirjian, A., Goldstein, H. & Tanner, J. M. A new system of dental age assessment. *Hum. Biol.* **45**(2), 211–227 (1973). 4714564

[10] Kreitner, K. F., Schweden, F. J., Riepert, T., Nafe, B. & Thelen, M. Bone age determination based on the study of the medial extremity of the clavicle. *Eur. Radiol.* **8**(7), 1116–1122 (1998). 9724422 10.1007/s003300050518

[11] Bassed, R. B., Drummer, O. H., Briggs, C. & Valenzuela, A. Age estimation and the medial clavicular epiphysis: Analysis of the age of majority in an Australian population using computed tomography. *Forensic. Sci. Med. Pathol.* **7**(2), 148–154 (2011). 21057985 10.1007/s12024-010-9200-y

[12] Hillewig, E. et al. Magnetic resonance imaging of the medial extremity of the clavicle in forensic bone age determination: A new four-minute approach. *Eur. Radiol.* **21**(4), 757–767 (2011). 20890759 10.1007/s00330-010-1978-1

[13] Schulz, R. et al. Studies on the time frame for ossification of the medial epiphysis of the clavicle as revealed by CT scans. *Int. J. Legal Med.* **119**(3), 142–145 (2005). 15711799 10.1007/s00414-005-0529-9

[14] Kasper, K. A., Austin, D., Kvanli, A. H., Rios, T. R. & Senn, D. R. Reliability of third molar development for age estimation in a Texas Hispanic population: A comparison study. *J. Forensic. Sci.* **54**(3), 651–657 (2009). 19432741 10.1111/j.1556-4029.2009.01031.x

[15] Levesque, G. Y., Demirijian, A. & Tanguay, R. Sexual dimorphism in the development, emergence, and agenesis of the mandibular third molar. *J. Dent. Res.* **60**(10), 1735–1741 (1981). 6944337 10.1177/00220345810600100201

[16] Olze A, Reisinger W, Geserick G, Schmeling A. Age estimation of unaccompanied minors. Part II. Dental aspects. Forensic Sci Int. 159 Suppl 1:S65–67 (2006). 10.1016/j.forsciint.2006.02.018 16529893

[17] De Angelis, D. et al. Application of age estimation methods based on teeth eruption: How easy is Olze method to use?. *Int. J. Legal Med.* **128**(5), 841–844 (2014). 24781787 10.1007/s00414-014-1006-0

[18] Thevissen, P. W., Pittayapat, P., Fieuws, S. & Willems, G. Estimating age of majority on third molars developmental stages in young adults from Thailand using a modified scoring technique. *J. Forensic. Sci.* **54**(2), 428–432 (2009). 19187460 10.1111/j.1556-4029.2008.00961.x

[19] Kellinghaus, M., Schulz, R., Vieth, V., Schmidt, S. & Schmeling, A. Forensic age estimation in living subjects based on the ossification status of the medial clavicular epiphysis as revealed by thin-slice multidetector computed tomography. *Int. J. Legal Med.* **124**(2), 149–154 (2010). 20013127 10.1007/s00414-009-0398-8

[20] Knell, B., Ruhstaller, P., Prieels, F. & Schmeling, A. Dental age diagnostics by means of radiographical evaluation of the growth stages of lower wisdom teeth. *Int. J. Legal Med.* **123**(6), 465–469 (2009). 19241087 10.1007/s00414-009-0330-2

[21] Jayaraman, J., Mendez, M. J. C., Gakunga, P. T. & Roberts, G. Age estimation of Hispanic children in the United States: Development and validation of dental reference dataset based on two staging systems. *Leg. Med. (Tokyo).* **56**, 102033 (2022). 35151981 10.1016/j.legalmed.2022.102033

[22] Riley, R. D. et al. Minimum sample size for external validation of a clinical prediction model with a binary outcome. *Stat. Med.* **40**(19), 4230–4251 (2021). 34031906 10.1002/sim.9025

[23] Khamis, H. Measures of association How to choose?. *J. Diagn. Med. Sonogr.* **24**(3), 8 (2008).

[24] Robin, X. et al. pROC: An open-source package for R and S+ to analyze and compare ROC curves. *BMC Bioinform.* **12**, 77 (2011). 10.1186/1471-2105-12-77 PMC3068975 21414208

[25] Team RC. R: A language and environment for statistical computing. *R Foundation for Statistical Computing* 2023 (Austria, 2023).

[26] Kuhn, M. Building predictive models in R using the caret package. *J. Stat. Softw.* **28**(5), 1–26 (2008).27774042

[27] Sarkar D. Lattice: Multivariate Data Visualization with R, (2008).

[28] Wickham H. Getting Started with ggplot2. ggplot2: Elegant Graphics for Data Analysis. Cham: Springer International Publishing;11–31 (2016).

[29] Kassambara A. ggpubr: ‘ggplot2’ Based Publication Ready Plots. (2023).

[30] Metz, C. E. Basic principles of ROC analysis. *Semin. Nucl. Med.* **8**(4), 283–298 (1978). 112681 10.1016/s0001-2998(78)80014-2

[31] Bassed, R. B., Briggs, C. & Drummer, O. H. The incidence of asymmetrical left/right skeletal and dental development in an Australian population and the effect of this on forensic age estimations. *Int. J. Legal Med.* **126**(2), 251–257 (2012). 21947631 10.1007/s00414-011-0621-2

[32] Kellinghaus, M. et al. Enhanced possibilities to make statements on the ossification status of the medial clavicular epiphysis using an amplified staging scheme in evaluating thin-slice CT scans. *Int. J. Legal Med.* **124**(4), 321–325 (2010). 20354711 10.1007/s00414-010-0448-2

[33] Meijerman, L., Maat, G. J., Schulz, R. & Schmeling, A. Variables affecting the probability of complete fusion of the medial clavicular epiphysis. *Int. J. Legal Med.* **121**(6), 463–468 (2007). 17909834 10.1007/s00414-007-0189-z PMC2039830

[34] Angelakopoulos, N. et al. Comparison of the third molar maturity index (I(3M)) between left and right lower third molars to assess the age of majority: A multi-ethnic study sample. *Int J Legal Med.* **135**(6), 2423–2436 (2021). 34228192 10.1007/s00414-021-02656-2

[35] Dreizen, S., Parker, G. S., Snodgrasse, R. M., Spies, T. D. & Webbpeploe, H. Bilateral symmetry of skeletal maturation in the human hand and wrist. *AMA J. Dis Child.* **93**(2), 122–127 (1957). 13393949 10.1001/archpedi.1957.02060040124004

[36] Anderson, M. Use of the Greulich-Pyle “Atlas of Skeletal Development of the Hand and Wrist” in a clinical context. *Am. J. Phys. Anthropol.* **35**(3), 347–352 (1971). 4332699 10.1002/ajpa.1330350309

[37] Tyr, A., Heldring, N. & Zilg, B. Examining the use of alternative light sources in medico-legal assessments of blunt-force trauma: A systematic review. *Int. J. Legal Med.* **138**(5), 1925–1938 (2024). 38844617 10.1007/s00414-024-03262-8 PMC11306313

[38] Heldring, N., Larsson, A., Rezaie, A. R., Råsten-Almqvist, P. & Zilg, B. A probability model for assessing age relative to the 18 year old threshold based on magnetic resonance imaging of the knee combined with radiography of third molars in the lower jaw. *Forensic. Sci. Int.* **330**, 111108 (2022). 34826761 10.1016/j.forsciint.2021.111108

[39] De Tobel, J. et al. Dental and skeletal imaging in forensic age estimation: Disparities in current approaches and the continuing search for optimization. *Semin. Musculoskelet Radiol.* **24**(5), 510–522 (2020). 33036039 10.1055/s-0040-1701495

